# Leveraging mobile health applications to improve sexual and reproductive health services in Nigeria: implications for practice and policy

**DOI:** 10.1186/s12978-021-01069-z

**Published:** 2021-01-23

**Authors:** Akaninyene Otu, Ido Ukpeh, Okey Okuzu, Sanni Yaya

**Affiliations:** 1 Department of Internal Medicine, College of Medical Sciences, University of Calabar, Cross River State, Calabar, Nigeria; 2 InStrat LLC, Montclair, NJ USA; 3 InStrat Global Health Solutions, Abuja, Nigeria; 4 School of International Development and Global Studies, Faculty of Social Sciences, University of Ottawa, 120 University Private, Ottawa, ON K1N 6N5 Canada; 5 The George Institute for Global Health, Imperial College London, London, UK

## Background

Universal access to sexual and reproductive health (SRH) is crucial for human well-being and is essential to achieve the sustainable development goals (SDG’s) [1]. SRH remains a major healthcare challenge facing Nigeria with evidence of marked inequities in access to SRH services that highlight a wide gap between rich and poor [2]. Nigeria, with an estimated population of 203 million, is the seventh-most populous country in the world [3] and has the fastest growing mobile market on the African continent [4]. Mobile phone subscription in Nigeria reached 180 million in October 2019 with phone penetration (tele density) being as high as 94.5% [5]. Smart phone usage in Nigeria is currently estimated at 40 million and is projected to rise to 140 million by 2025 [6]. The increasing penetration and use of mobile phones in developing country settings such as Nigeria has led to greater utilization of mobile health (mHealth) which involves the incorporation of mobile phones, tablets and other wireless devices to support the achievement of health objectives [7]. According to the World Health Organization (WHO), mHealth has the potential to transform how people access health services and achieve higher standard of health and well-being [8].

Evidence from other settings suggest that mHealth can be used to bridge the gaps of distance and expertise and support SRH services such as antiretroviral adherence [9], the prevention of sexually transmitted infections [10, 11] and perinatal and postnatal care [12]. Despite the growing body of evidence to support the use of mHealth to provide SRH, implementation of these mHealth solutions remains surprisingly low in developing countries [13]. With high mobile phone penetration in Nigeria, the adoption of mHealth to transform SRH services appears to be low-hanging fruit that should be fully exploited.

## The fragile state of SRH in Nigeria

While progress has been recorded with promoting SRH services in Nigeria and reducing maternal mortality rates, the country accounts for nearly 20% of all global maternal deaths [14] with an estimated maternal mortality ratio (MMR) of 917 deaths per 100,000 live births (uncertainty interval 80%: 658–1320). Between 2005 and 2015, over 600,000 maternal deaths are estimated to have occurred in Nigeria with the lifetime risk of dying during pregnancy, childbirth or postpartum/post-abortion being put at 1 in 22 [14]. These unacceptably high rates are a reflection of inequities in access to health services with poor women in remote areas being the least likely to access reproductive healthcare services [7]. In 2020, over 10 million pregnancies are projected to have occurred in Nigeria with 65% of these estimated to have happened in rural (and mostly hard-to reach) areas [15]. Low numbers of skilled birth attendants, especially in remote areas, have resulted in many unsafe births in Nigeria. Between 2006–2014, it is projected that just 43% of births in Nigeria were attended by skilled birth attendants [15].

With a life expectancy of 55 years, Nigeria’s population is a very youthful one with approximately 31.9% of the population falling within the 12–24-year age bracket. The contraceptive prevalence rate among Nigerian women aged 15–49 is estimated to be 17% with an unmet need for family planning rate of 15%, this is against the backdrop of a high total fertility rate of 5.2% per woman [15]. A high prevalence of child marriage exists in Nigeria as approximately 43% of girls are married by age 18 with higher rates of sexually transmitted infections (such as HIV) and complications during pregnancy and childbirth occurring in this age group [15]. Married girls appear to be further disadvantaged as only 2% percent of 15–19-year-old married girls are projected to be in school and receiving an education [16]. More so, female genital mutilation prevalence among girls aged 15–19 in Nigeria is estimated to be 14%.

The SRH coverage rates in Nigeria are typically low, especially in the rural (and hard-to-reach) areas and among adolescents [17, 18]. Some of the identified barriers to accessing SRH services include inadequate knowledge about the services, inconvenient health facility opening hours, remoteness of facilities, financial constraints as well as community and religious norms [19]. Wider systemic issues also impact negatively on the SRH system in Nigeria. Healthcare financing in Nigeria is grossly inadequate and contributes greatly to the fragility of the health system. Since the Abuja declaration of 2001 that saw leaders of African countries pledge to commit at least 15% of their annual budgets to the health sector, Nigeria has never voted more than 6% of its annual budget to the health sector. To further compound this situation, household out-of-pocket expenditure remains by far the largest source of health expenditure as less than 5% of Nigerians are covered by the National Health Insurance Scheme (NHIS) [20]. This has probably led to highly convoluted patterns of care-seeking.

## Emergence of a digital technology culture: transforming traditional healthcare

mHealth is the first component of electronic health (eHealth); eHealth refers to use of information and communication technologies (ICTs) for health and health-related fields which reaches hard-to-access areas [21]. The last few decades have recorded a steady rise in the utilization of eHealth to strengthen health systems. In 2005, The World Health Assembly urged member states to consider developing and implementing eHealth to promote equitable and affordable health access for their citizens [22]. In 2013, the World Health Assembly adopted a resolution on eHealth standardization and member countries were encouraged to establish a national eHealth strategy [22]. In 2015, the United Nations General Assembly on Information Society declared that widespread adoption of eHealth could facilitate the achievement of the Sustainable Development Goals (SDGs) in 2030 [23]. In 2019, the WHO released its first guidelines on eHealth interventions. Despite the efforts of international organizations to promote eHealth, Africa appears to be lagging behind in the adoption of this valuable resource to strengthen its health services.

With technological innovations becoming increasingly inseparable from healthcare, there are concerns of an imminent paradigm shift involving patients (who now have greater access to unregulated technological solutions to deal with their health conditions) and health workers operating within inflexible health systems that have failed to catch up with the rapid progress of the medical technology industry [24].

The advances within the medical technology industry do not appear to be matched by changes within the medical curriculum or policies and guidelines behind medical care with potential negative impacts on quality of care [25]. Strict regulations within healthcare systems; hesitation among stakeholders in healthcare to implement change; cultural and human factors are some identified reasons for the slow transition of health systems to eHealth. This is occurring in the setting of increasing costs of caring for people with chronic conditions and imminent health worker shortages globally. Expanded access to electronic medical information has given rise to empowered “e-patients” who take active part in decision making about their own health and disease management [26]. This has resulted in a shift from the patriarchal hierarchy of traditional medicine where health workers were responsible for making medical decisions with minimal input from patients to an equal level partnership between patients and professionals [27]. In proposing the use of mHealth to deliver SRH services in developing country settings such as Nigeria, a model that reflects this shift from the old paradigm of the paternalistic model of medicine is desirable.

Emerging evidence has linked the perennial problems of rapid population growth, high birth rates and dismal rates of maternal mortality and HIV/AIDS in the sub-Saharan Africa with a distinct pattern of sexual behavior among adolescents. In spite of a growing commitment by national and international actors to address this peculiar issue, several political, economic, cultural and religious barriers have limited progress on this front [28]. Understanding the nuances that exist in adolescent SRH in sub-Saharan Africa will be key to designing appropriate and effective mHealth interventions for SRH in the region.

## Utility of mHealth in improving SRH services

Advances in mHealth have promoted their use in SRH service delivery. mHealth has been utilized to send personal reminders to patients via e-mail, automated phone calls, or text messages to remind them to take their medications or visit the health facility [29]. Text message and telephone reminders have been successfully used to gather health‐related information from pregnant women [30], to increase access to family planning information [31], to facilitate the presence of skilled birth attendants during labour [32], and to enhance prenatal and postnatal care [12]. A recent systematic review revealed that an SMS-reminder intervention improved medical compliance (e.g., taking medication at a scheduled time, completing non-medication follow-up treatment) in 85% (83/97) of the studies considered [33]. The effectiveness of SMS use for SRH services has been associated with the women's digital literacy levels and their knowledge of the local language in text [34].

Mobile gadgets have caused a shift in the practice of medicine from the traditional doctor-patient meeting to electronic-aided communication and procedures. Consultation by telephone helps to reduce waiting times in consultation rooms, increase frequency of contact, enhance communication between caregiver and patients thereby building confidence and rapport, reduce visits to emergency room and reduce travel time and transportation cost [35, 36]. The utility of mobile consultations on telephone or via platforms such a Microsoft Teams, Zoom and Webex is further underscored by the current COVID-19 pandemic where social distancing measures are critical to reducing the spread of virus in clinical settings. These social media platforms are acceptable to a majority of the younger generation who form the bulk of clients requiring SRH services and should be exploited for this purpose.

In Morocco, the effectiveness of portable ultrasound machines and 3G smartphones in improving diagnostic times for expectant mothers in rural clinics has been clearly demonstrated. These devices were wirelessly linked to reproductive health specialists in urban hospitals [37]. Smartphone connected electrocardiograms (ECG) are now a reality and smartphones have been successfully used to monitor heart rate, blood oxygen saturation, and blood pressure [38].

mHealth has been used to train SRH e healthcare workers in remote settings and provide them access to accurate and current clinical information which they could review at their convenience on their mobile devices. Previous research has demonstrated the effectiveness and feasibility of using mHealth to train frontline health workers in rural settings within Nigeria [39]. Patients have also been empowered by providing them with accurate and readily available information on SRH on their mobile devices. mHealth has been shown to improve healthcare worker productivity in several ways: (1) reducing unproductive travel time, (2) enabling faster decision-making by improving knowledge and care giving skills, (3) improving communications, (4) promoting rapid response to medical test results that are transmitted directly to mobile phones, (5) improving data management and record-keeping practices, (6) reducing  medication errors, (7) enhancing overall professional efficiency and work patterns [29, 40, 41]. These benefits could be extended to healthcare workers in SRH upon adoption of mHealth approaches.

## Applicability of mHealth to the Nigerian context: implications for practice and policy

Is mHealth really suitable for the Nigerian health system? A recent survey conducted by InStrat Global Health Solutions in 339 health facilities across Nigeria (with representative geographic coverage of Nigeria) revealed that there was an average of 7 phones per facility of which 5 were smart phones capable of supporting most health technology applications [42]. This supports the projections of high mobile telephone penetration in Nigeria and lends plausibility to the theory that mHealth can be used to strengthen SRH services in Nigeria and indeed other similar contexts. Local examples of the utility of mHealth in improving SRH services exist. Ondo State Nigeria had the highest MMR in the southwest region of 775 per 100,000 live births in 2008 [43]; up to 90% of these deaths were attributed to the activities of untrained birth attendants. An electronic tablet-based training intervention by InStrat GHS consisting of short films (in both English and Yoruba) interspersed with quizzes on maternal care, basic emergency obstetric and newborn care resulted in a 32% improvement in the test scores of the 200 health workers trained [44].

The huge challenges posed by the unmet SRH needs of Nigerians call  for the full-scale adoption of an innovative and cost-effective measure such as mHealth. Technological and policy changes will be required to adapt mHealth to suit the Nigerian SRH context. Mobile phones are a useful tool for generating self-entered and automatically captured data on SRH but centralized databases will need to be created instead of isolated systems [45, 46]. Coordination will be required to synchronize the development of software on SRH to guarantee functionality on different operating systems [47]. Funding will need to be secured to cover initial software development, routine system troubleshooting and ongoing costs of internet and mobile network for health workers. mHealth remains an affordable solution as the only requirement from the participants is a mobile phone. The provision of toll-free hotlines for SRH has found to be beneficial in sub-Saharan settings and should be explored within the Nigerian context. Extensive training and supportive supervision are crucial; cultural and language adaptation are vital as demonstrated in the successful maternal care training that was conducted in Ogun State. Data security is a key consideration as electronic systems are vulnerable to hacking and data theft. Mitigation measures should be put in place to protect electronic patient records and sensitive information. Electronic files should be backed up to reduce loss in case of fire or other physical disasters [48].

mHealth holds great promise for revolutionizing the delivery of SRH services within Nigeria and similar contexts. To realize the full potential of mHealth, stakeholders will be required to carefully address the potential drawbacks to its full-scale deployment.

## Data Availability

Not applicable.

## Abbreviations

ICTs: Information and Communication Technologies
mHealth: Mobile Health
SDGs: Sustainable Development Goals
SRH: Sexual and Reproductive Health
WHO: World Health Organization
